# Commentary to “Morphometry and Contents of the Suprascapular Notch with Potential Clinical Implications: A Cadaveric Study”

**DOI:** 10.1055/s-0042-1747960

**Published:** 2022-06-20

**Authors:** Azzat Al-Redouan, David Kachlik

**Affiliations:** 1 Department of Anatomy, Second Faculty of Medicine, Charles University, Prague, Czech Republic

We read with interest the article “Morphometry and contents of the suprascapular notch with potential clinical implications: a cadaveric study” by Tsikouris et al. 1 However, we would like to point out several data that we find contradictory to our findings in previous studies and we have differing point of view.

The aforementioned study brought up an interesting hypothesis which discussed whether there is a correlation of an ossified superior scapular transverse ligament, also called suprascapular ligament (SL), 2 to a dimensioned middle-transverse diameter of the suprascapular notch (SSN) in the SSN Type-IV according to Polguj et al SSN morphometric classification, 3 which is also referred to as suprascapular foramen. 2 The presented study suggested that an ossification process in the SL was correlated to SSN space narrowing in its horizontal plane and contributing to suprascapular nerve (SN) compression, but this premise does not seem to be the case. A SSN with a middle-transverse diameter mean of 5.10 mm can still accommodate the passing SN. The study by Tubbs et al demonstrating a compressed SN in 5 SSN out of 50 cadaveric studies was evidenced by histolopathological examination of the SN, and the diameter of those SSN was at critical stenosed range of 1.8 to 3.0 mm. 4 That make the study by Tubbs et al 4 case specific and not necessary cohort in the absence of SSN stenosis. Meanwhile, Tsikouris et al 1 demonstrated smaller in size SSN in association to SL ossification in their population sample but those SSN were not critically stenosed.

The focus on parameter in the study by Tsikouris et al 1 was the middle-transverse diameter of the SSN. This parameter when reduced beyond its critical point leads to a horizontal type of a stenosis which is between the medial and lateral bony margins of the SSN and not bordered by the SL. 5 And this type of stenosis was also found in SSN Type-I (deep notch) prominently and in combined form in SSN Type-II (depth equal to width notch) and to a lesser extent in SSN Type-III (wide notch). 5 On the other hand, SSN Type-IV (foramen) which carries the ossified SL, takes the form of a circular stenosis which is also dependent on the SSN height and not merely on the SSN middle width. 5 In addition, the SSN takes prominently a U-shape or V-shape in terms of its inferior margin where the medial and lateral margins meet. Therefore, a reduced middle-transverse diameter does not entitle a reduced superior-transverse diameter of the SSN. 5 This makes the middle-transverse diameter not a candidate parameter to be selected in isolation to other parameters.

The second addressed point in the article by Tsikouris et al 1 was concerning the number of passing vessels within the SSN according to the morphological classification by Polguj et al. 6 Tsikouris et al concluded that three elements passing through the SSN would cause SN nerve compression. 1 However, the diameters of the passing vessels in relation to the SSN vertical and horizontal parameters were not elaborated on. In fact, an accompanying suprascapular vein can give protective properties by serving as a cushion against the SSN bony margins. 7 8

In conclusion, the ossified SL does not necessary reduce the SSN internal space to a critical size. Type-V (the discreet notch) followed by Type-III (width larger than height) showed higher incidence of stenosis than Type-IV (foramen variant). 5 Ossification of the SSN margins has more role in reducing the space capacity than ossification of the SL. Nevertheless, a nonossified SL can be flat in shape with sharper edge that can cause sling-effect irritation to the SN. 8 A passing vessel would generate risk of stenosis if it reduced the SSN space capacity beyond an accommodating space for the passing SN.
